# A comparison of two group cognitive behavioral therapy protocols for anxiety in urban schools: appropriateness, child outcomes, and cost-effectiveness

**DOI:** 10.3389/fpsyt.2023.1105630

**Published:** 2023-06-23

**Authors:** Gwendolyn M. Lawson, Abbas F. Jawad, Rachel Comly, Muniya Khanna, Henry A. Glick, Rinad S. Beidas, Jessica Goldstein, Shelby Brizzolara-Dove, Tara Wilson, Quinn Rabenau-McDonnell, Ricardo Eiraldi

**Affiliations:** ^1^Children’s Hospital of Philadelphia, Philadelphia, PA, United States; ^2^Perelman School of Medicine, University of Pennsylvania, Philadelphia, PA, United States; ^3^OCD and Anxiety Institute, Bryn Mawr, PA, United States; ^4^Department of Medical Social Sciences, Feinberg School of Medicine Northwestern University, Chicago, IL, United States

**Keywords:** anxiety, CBT, school, implementation, cost

## Abstract

**Background:**

Cognitive behavioral therapy (CBT) for pediatric anxiety is efficacious for reducing anxiety symptoms and improving functioning, but many children are unable to access CBT for anxiety in community settings. Schools are an important setting in which children access mental health care, including therapy for anxiety. In this setting, therapy is usually delivered by Masters-level therapists.

**Objectives:**

Friends for Life (FRIENDS), a 12-session, manualized, group CBT program for anxiety has demonstrated effectiveness when implemented in schools. However, prior research has also found challenges regarding feasibility and cultural fit when delivering FRIENDS in the urban school context. To address these challenges, we adapted FRIENDS for implementation in the school setting so that it might be more feasible and culturally appropriate for low-income, urban schools in the United States, while maintaining the core components of treatment. The current study uses a mixed-method approach to compare the effectiveness, cost-effectiveness, and perceived appropriateness of FRIENDS and CATS when delivered by Masters-level therapists with train-the-trainer support.

**Materials and methods:**

First, we compared change scores for student outcomes (i.e., child-report MASC-2 total score, parent-report MASC-2 total score, teacher-report Engagement and Disaffection subscale scores) from pre- to post- treatment between students receiving FRIENDS and students receiving CATS to assess whether the two conditions resulted in equivalent outcomes. Second, we compared the cost and cost-effectiveness between the groups. Finally, we used an applied thematic analysis to compare appropriateness of the interventions as perceived by therapists and supervisors.

**Results:**

The mean change score for the child-reported MASC-2 was 1.9 (SE = 1.72) points in the FRIENDS condition and 2.9 (SE = 1.73) points in the CATS condition; results indicated that the conditions were similar in their treatment effects, and symptom reductions were small in both groups. The modified protocol, CATS, was shown to cost significantly less to implement compared to FRIENDS and showed greater cost-effectiveness. Finally, compared to therapists and supervisors in the CATS condition, therapists and supervisors in the FRIENDS condition more strongly described aspects of the intervention that were not appropriate for their context and in need of more extensive adaptations.

**Conclusion:**

Relatively brief, group CBT for anxiety, with adaptations to improve cultural fit, is a promising approach to treat youth anxiety symptom when delivered by school-based therapists with train-the-trainer implementation support.

## Introduction

Anxiety disorders are prevalent (1) and impairing (2) among children and adolescents, which makes untreated anxiety in youth an important public health problem. Fortunately, cognitive behavioral therapy (CBT) for pediatric anxiety is efficacious for reducing anxiety symptoms and improving functioning in both individual (3, 4) and group (5) therapy models. Cognitive behavioral therapy for anxiety typically includes core components such as exposure, cognitive restructuring, and psychoeducation, although there can be variability in the application of core components across CBT protocols (6).

Despite the existence of efficacious and effective treatments, the majority of youth with or at risk for anxiety disorders do not receive treatment of any kind. For example, in one nationally representative sample, only 1 in 5 adolescents with anxiety disorders reported receiving treatment for anxiety within their lifetime (7). Furthermore, youth who receive treatment commonly do so in community settings, in which clinician uptake of CBT is often low (8). A number of factors may contribute to this low uptake, including lack of resources and training opportunities (9), the cost for agencies and systems (10), and clinicians perceiving CBT components as unacceptable or inappropriate for their clients (11). Limited access to CBT for anxiety in community settings is particularly concerning because it may contribute to racial and socioeconomic inequities in treatment outcomes (12).

One promising approach to increase access to CBT for anxiety, particularly among youth from minoritized and marginalized backgrounds, is to provide services in the school setting. The provision of services within schools can help minimize many barriers that families face accessing treatment in traditional outpatient or hospital settings (13, 14) and may promote generalizability of skills practice (15). Additionally, using an indicated prevention model (i.e., providing CBT to children and youth who have symptoms of anxiety, but do not necessarily meet full criteria for an anxiety disorder) has the potential to benefit youth who are at risk for an anxiety disorder and decrease the public health burden of anxiety (16). Finally, providing treatment in a group, rather than individual, format may help increase intervention reach because the group format enables a single therapist to treat several children at once.

Friends for Life (FRIENDS; 17) is a manualized, group CBT program for anxiety in youth that has demonstrated effectiveness when implemented in schools as a universal prevention program, selective prevention program, and intervention for youth with anxiety disorders [e.g., (18–20)]. The FRIENDS program was adapted from *The Coping Cat* (21), a well-established empirically supported intervention for treating anxious children (22, 23).

Although effective and tested for use in schools, there are several challenges of feasibility and contextual appropriateness when delivering Friends for Life in the urban school context. First, the FRIENDS protocol consists of 10 weekly 90-min sessions plus 2 booster sessions. The typical class period for K-8 schools in the United States are between 40 and 50 min in duration. Dedicating 90-min per week for 12 weeks for an indicated prevention, group intervention would detract significantly from instructional time and would not be feasible for students in most school settings. Second, the FRIENDS protocol was designed for students in Australia and uses language, metaphors, and examples specific to the Australian school context (e.g., reference to a character who loved being able to go to the rainforest for walks).

To address these challenges, we adapted FRIENDS for implementation in the school setting so that it might be more feasible and culturally appropriate for low-income, urban schools in the United States. We followed procedures developed by Bernal et al. (24), including surveying service providers and trainers regarding the appropriateness of content for the target population. The CBT for Anxiety Treatment in Schools (CATS) (25) was designed as an 8, 35-min session manual that includes the 5 essential components of CBT for anxiety as in *Coping Cat*: psychoeducation, somatic management skills training, cognitive restructuring, exposure, and relapse prevention plans. The CATS manual follows the school-based group CBT format of FRIENDS and is designed to be feasible for implementation in the United States public school setting, particularly urban schools serving a predominantly minoritized and low-income population. We also made changes to the language and specific examples used, in order to improve the cultural appropriateness within the United States urban school context, and changed session activities in order to make the group sessions more engaging.

Given the current public health burden of untreated anxiety, it is critical to identify prevention and treatment approaches that are effective for youth, and are also feasible and contextually appropriate for usual care settings. Because the CATS program retains the core components of an evidence-based manualized treatment and has been modified to improve contextual appropriateness and feasibility, it may meet this need for United States urban school settings. In order to determine its promise, it is important to empirically examine the effectiveness and perceived contextual appropriateness of the modified CATS protocol, compared to FRIENDS, when delivered in United States urban schools.

Moreover, given the financial pressures faced by the mental health agencies that provide school-based services (26), it is also important to understand the cost of implementing particular therapy protocols in this setting. The cost of an intervention and implementation approach is important to the implementation and sustainment of any given intervention (27) and may be particularly important in the public mental health sector (10). Cost-effectiveness evaluations can inform intervention and implementation strategy selection by examining implementation costs in relation to health outcomes (28), such as child anxiety symptoms. It is therefore also important to compare the cost and cost-effectiveness of the two anxiety treatment protocols.

### Current study

The current study was conducted as part of a larger Hybrid Type II effectiveness implementation trial of group CBT for anxiety in urban schools (29). In the larger study, school-based therapists and their supervisors were randomized to deliver one of two group intervention models (i.e., FRIENDS or CATS), and those who delivered CATS were also randomized to the type of implementation support they received. The current study focuses on the comparison between children and therapists/supervisors randomized to the FRIENDS condition and those randomized to the CATS condition, both receiving the same type of train-the-trainer implementation support.

In this paper, we address three related aims regarding the comparison between the FRIENDS and CATS interventions. First, we compared mean differences across time in child-reported, parent-reported, and teacher-reported outcomes between the two groups to determine whether there were clinically significant differences in outcomes between CATS and FRIENDS. Second, we compared the cost of implementing the two interventions and their cost-effectiveness. Finally, we used a qualitative approach to compare appropriateness of the interventions as described by therapists and supervisors in semi-structured qualitative interviews. Taken together, the current study contributes to the knowledge base about the implementation of CBT for anxiety in school settings by using multiple methods to examine the promise of a group CBT intervention with adaptations to improve cultural fit delivered by school-based therapists within the context of urban schools.

## Materials and methods

### Procedures

All procedures were approved by the school district research board and the Philadelphia Department of Public Health research board (FWA00003616). Data were collected as part of a cluster randomized, hybrid effectiveness-implementation trial. We recruited public mental health agencies that provide prevention and treatment services in public and charter schools for participation. A total of nine agencies agreed to participate. After agreeing to participate, agency administrators were asked to provide a list of schools where their agency provided mental health services that they thought would be good candidates for participation. Following the initial school selection, investigators obtained consent for their schools’ participation from school principals. A total of 36 public and charter schools participated in the larger study. Randomization occurred at the school level, stratified on school size.

Therapists and supervisors in participating agencies and schools consented to participation, and nominated students in grades 4–8 for participation. Nominated students were already enrolled in the mental health program at their school. Students were screened for participation using the Screen for Anxiety Related Disorders (SCARED; 30); in order to participate, they were required to score above criteria on the Total score (i.e., ≥25), and/or one of the following subscale scores: Panic Disorder or Significant Somatic Symptoms (i.e., ≥7), Generalized Anxiety Disorder (i.e., ≥9), Separation Anxiety Disorder (i.e., ≥5) and/or Social Anxiety Disorder (i.e., ≥8) on either the child-report or parent-report SCARED.

See Eiraldi et al. (29) for a full description of recruitment and randomization procedures.

### Train-the-trainer implementation support

Participating therapists in both conditions received ongoing support from a clinical supervisor employed by their agency, with whom they were expected to meet for weekly supervision as part of their study participation. Supervision sessions were expected to include time preparing for the upcoming session, as well as performance feedback. Supervisors in both conditions received initial training from the research team prior to the initiation of the groups (i.e., Train-the-Trainer implementation support). This training focused on supervision best practices (31), as well as CBT principles, best practices for managing groups, and content and procedures for specific group manual (i.e., CATS or FRIENDS). The training lasted approximately 8 h and was sometimes split into several sessions, as needed.

Additionally, prior to beginning the groups, therapists received an initial training from the research team focused on knowledge and skills regarding the group CBT manual they were randomized to implement. The training lasted approximately 8 h and was sometimes split into several sessions, as needed. See Eiraldi et al. (29) for a full description of implementation support.

### Participants

#### Student sample for effectiveness analyzes

The full analysis set (FAS) for the effectiveness analyzes was defined to include all student participants who (a) have assessment data at baseline for at least one of the primary measures and were randomized into treatment condition; (b) received at least one session of therapy; and (c) have post- assessment data for at least one of the primary outcome measures. Additionally, to account for the cluster-randomized design, we also imposed the following criterion for inclusion in the FAS: (d) the student’s school includes at least 3 student participants who meet the above criteria. The FAS consists of 91 students (i.e., 48 students who received FRIENDS and 43 students who received CATS) from 19 schools.

In the broader sample, there were a total of 158 student participants who were randomized to one of the relevant conditions and provided baseline measurements for a primary outcome (i.e., 75 students in the FRIENDS condition with standard Train-the-Trainer support and 83 in the CATS condition with standard Train-the-Trainer support). Of these participants, 120 (i.e., 59 FRIENDS and 61 CATS) received at least one session of therapy. Of this group of participants, 97 also provided post-baseline assessment data for at least one of the primary outcome measures. Of these participants, 91 students met the criteria of their school including at least 3 student participants who met criteria and were included in the FAS.

#### Sample for cost-effectiveness analyzes

The sample for cost-effectiveness analyzes was defined at the level of group cohorts (i.e., the therapist, supervisor, and students who participated in a FRIENDS or CATS group; in some cases, therapists and/or supervisors led multiple cohorts, although no students participated in more than one cohort). Cohorts were included if they met the following criteria: (a) at least one student within the cohort contributed child-report MASC-2 data, (b) at least one therapist or one supervisor reported time tracking data. Data for cost-effectiveness analyzes were available from 28 cohorts from 21 schools. Twenty-four therapists contributed at least one estimate of weekly time data, with an average of 2.5 estimates per therapist; 22 supervisors contributed at least one estimate of weekly time data with an average of 2.6 estimates per supervisor. Eighty-three students from 18 schools contributed child-report MASC-2 scores.

#### Therapists and supervisors for qualitative analyzes

Fifteen therapists and 14 supervisors participated in the CATS condition and 11 therapists and 11 supervisors participated in the FRIENDS condition. Of those therapists and supervisors, 21 therapists (10 CATS and 11 FRIENDS) and 21 supervisors (11 CATS and 10 FRIENDS) completed semi-structured qualitative interviews. The sample of supervisors was 79% female; 71% of supervisors were Black, 21% were White, 8% did not report their race; 8% were Hispanic. Nearly all (92%) supervisors’ highest education level was a Master’s degree, and 2 supervisors had a Doctorate. The sample of therapists was 91% female; 55% of therapists were Black, 41% were White, 4% did not report their race; 0% were Hispanic. All therapists had a Master’s degree.

### Measures

#### Effectiveness outcome measures

**Multidimensional Anxiety Scale for Children – 2^nd^ Edition (MASC-2)**. The MASC- 2 is a 50-item, 4-point rating scale (0 = never; 1 = rarely; 2 = sometimes; 3 = often) organized around six subscales and a Total score used for the assessment of anxiety symptoms in children. The instrument has strong psychometric properties, and it is effective for measuring treatment effects (March, 2012). It showed excellent internal consistency in the full baseline sample (*α* = 0.91 for child self-report; *α* = 0.92 for parent-report MASC-2). We used the child self-report and parent-reported Total T Scores in analyzes.

**Engagement versus Disaffection with Learning-Teacher Report (EvsD-Teacher)**. The EvsD-Teacher is a teacher-reported, 20-item, four-point (1 = not at all true; 2 = not very true; 3 = sort of true; 4 = very true) instrument with four sub-scales: (a) *Behavioral Engagement*, (b) *Emotional Engagement*, (c) *Behavioral Disaffection,* and (d) *Emotional Disaffection* (32). The subscales measure behavioral and emotional academic engagement and disaffection, as reported by teachers. Internal consistency of the subscales in the full baseline sample ranged from *α* = 0.73 for Emotional Disaffection to *α* = 0.90 for Emotional Engagement. We used the average score for each of the four scales in analyzes.

#### Implementation outcome measures

##### Fidelity measurement

Content fidelity of group treatment sessions in both conditions was measured by coding video recordings of group treatment sessions using a Content Fidelity Checklist (33) in which raters use a yes/no response scale to indicate whether or not the implementer covered each component from the manual in the group session. Content fidelity scores were computed as the percentage of components that were covered. Approximately 75% of sessions were selected for coding, and approximately 39% of those sessions were re-coded by a second rater for inter-rater reliability. The ICC (2,2) for the total percent of content covered was 0.62. See Eiraldi et al. (29) for a full description of training procedures and reliability monitoring procedures.

##### Dosage measurement

We used group attendance records to calculate the proportion of students in each condition who received at least one session, at least half of the group sessions, and all of the group sessions.

##### Cost measurement

In order to estimate the cost of implementing the FRIENDS and CATS groups, we measured the time that therapists and supervisors self-reported spending on activities related to group implementation and multiplied it times hourly wages. Clinicians and supervisors were asked to complete logs that recorded the time they spent on activities that we categorized under the headings of communication, consultation, group, preparation, supervision, screening, training, uploading material, and travel. Annual salaries plus benefits were derived from local community mental health agencies as well as public listings for relevant, local job postings. Hourly wages were derived based on an assumption of a 1920 (48 weeks * 40 h) hour work year. Wages were assigned to therapists and supervisors based on their years of experience.

##### Semi-structured qualitative interviews with therapists and supervisors

The semi-structured interview protocol was designed to explore therapists and supervisors’ perceptions of the group treatment sessions and the type of support that therapists and supervisors received. Relevant to the current analysis, the interview protocol for therapists included open-ended questions experiences leading groups (e.g., “Let us talk about when you actually ran the group. How did it go?”) and follow-up probes regarding the content of the group manual and any modifications (“Do you have any specific feedback about what is either working or not working well for your group when it comes to the content of the manual?,” “Did you make any changes or modifications to the content of the manual?”). Members of the research team (e.g., research assistants) conducted the interviews, with training and supervision from clinical psychologists from the research team. Qualitative interviews were audio-recorded, transcribed, and de-identified, and de-identified transcripts were used for coding and analyzes.

### Analytic procedures

#### Effectiveness of FRIENDS versus CATS

We estimated the 95% confidence limits for mean differences in child outcomes (i.e., child-report MASC-2 total score, parent-report MASC-2 total score, teacher-report Engagement and Disaffection subscale scores) from pre- to post- treatment between children receiving FRIENDS and children receiving CATS to test whether the interventions are equivalent regarding effect sizes for these outcomes. Based on the estimated 95% CI, the criteria for equivalence were predefined to have margin of error associate with the 95% CI = ± 4.2. Therefore, if the 95% confidence interval of the mean pre-to post differences between the two interventions includes Zero, we would conclude that the two interventions produced similar effects on child outcomes. However, if the 95% confidence intervals did not include zero and the upper/lower limit exceeds 4.20, then we will conclude that one condition is superior to the other. The 95% CI margin of error of 4.20 was chosen based on the distribution of the reported mean effect sizes associate with FRIENDS (19). Because students were nested within schools and randomization occurred at the school level, we used a mixed effect approach to account for the cluster randomized design, with students nested within their randomized school.

#### Cost-effectiveness

We estimated the cost per one point improvement in each cohort’s average child self-report MASC-2 score (i.e., the average for the children in the cohort, not for a single child in the cohort). The numerator of the incremental cost-effectiveness ratio was the difference in average costs per cohort between the 2 groups (i.e., CATS minus FRIENDS). Negative values for the numerator indicate that CATS reduced costs; positive values indicate the opposite. The denominator was the difference in average change in MASC-2 scores (i.e., CATS minus FRIENDS). For the latter, a 1-point larger decrease in the MASC-2 score was considered a 1-point improvement. In addition, we plotted the empirical joint distribution of the differences in costs and effects on the cost-effectiveness plane and used it to depict the 95% confidence interval for the cost-effectiveness ratio. We also plotted the cost-effectiveness acceptability curve, which reports the probability that a therapy is good value for varying values of willingness to pay for a 1-point improvement in MASC-2 scores.

#### Appropriateness

Semi-structured qualitative interview transcripts were coded in multiple stages (34) using an integrated inductive and deductive approach. Coding was conducted by the two research assistant coders (Bachelors- or Masters- level research assistants), supervised by a master coder (clinical psychologist). Approximately, 20% of the interviews were coded by all three coders and discussed at biweekly meetings throughout the coding process, and any disagreements were resolved by discussion. See Lawson et al. (35) for a full description of the codebook development and coding process.

A total of 46 interviews (therapists and supervisors in the FRIENDS TT and CATS TT condition) were used in the current analyzes. Excerpts coded as “Intervention/Curriculum” (“when the interviewee discusses the content of the intervention”) were used for the current analyzes. We used applied thematic analysis (36) to examine similarities and differences in themes regarding perceptions of the group intervention between the two conditions (FRIENDS TT and CATS TT) through a multiple-stage process. A member of the research team first prepared a concept-by-text matrix to organize coded text into themes for each condition (i.e., FRIENDS, CATS) and respondent group (i.e., supervisors, therapist). The team member then drafted a second-stage analytic memo which the master coder vetted (33). The second-stage memo included summaries and illustrative quotes of the themes that were observed in each condition and respondent group, as well as a comparison of themes that were similar or different between conditions. The analysis reported here focuses on the comparison of therapist and supervisor perceptions regarding the contextual appropriateness of the intervention between conditions.

## Results

### Effectiveness outcomes

#### Student baseline characteristics

Demographic characteristics and baseline assessment scores for students in the sample overall and in the FRIENDS and CATS conditions are displayed in Table 1. The overall sample of students was approximately 66% male and had a mean age of just under 11 years. The overall sample of students were majority (i.e., 68%) Black, and 34% of the overall sample was of Hispanic ethnicity. There were no significant differences between the groups on student age, student grade level, student gender, or student ethnicity. However, the FRIENDS condition had a significantly higher proportion of students who were Black (77%) compared to the CATS condition (57%; *p* = 0.02; See Table 1).

**Table 1:** Student baseline characteristics for total sample and comparison by group (*N* = 91).

Baseline characteristic	FRIENDS (*N* = 48)	CATS (*N* = 43)	Total (*N* = 91)	Test for group comparison	*p* value for group comparison
	N (%)/*M* (*SD*)	N (%)/*M* (*SD*)	N (%)/*M* (*SD*)		
Gender					
Male	31 (64.6%)	29 (67.4%)	60 (65.9%)	Fisher’s	0.83
Race (*N* = 86)					
Black	37	24	63	Chi-Square	0.04
White	5	8	13		
Multiple	0	5	5		
Unknown	2	3	5		
Hispanic ethnicity (*N* = 84)	11 (23.9%)	14 (35.0%)	25 (29.1%)	Fisher’s	0.34
Grade Level					
4^th^	23 (47.9%)	18 (41.9%)	41 (45.1%)	Chi-Square	0.42
5^th^	10 (20.8%)	11 (25.6%)	21 (23.1%)		
6^th^	3 (6.3%)	7 (16.3%)	10 (11.0%)		
7^th^	9 (18.8%)	4 (9.3%)	13 (14.3%)		
8^th^	3 (6.3%)	3 (7.0%)	6 (6.6%)		
Age	10.96 (1.58)	10.91 (1.49)	10.93 (1.53)	*t* test	0.87
Baseline assessment scores					
MASC-2 – Child report (*N* = 91)	58.35 (11.95)	58.56 (12.54)	58.45 (12.16)	*t* test	0.94
MASC-2 – Parent report (*N* = 36)	60.94 (13.24)	63.25 (17.81)	62.22 (15.77)	*t* test	0.67
EvsD – Behavioral Engagement (N = 89)	3.34 (0.62)	3.39 (0.62)	3.36 (0.62)	*t* test	0.73
EvsD – Emotional Engagement (N = 89)	3.06 (0.65)	3.02 (0.72)	3.04 (0.68)	*t* test	0.78
EvsD – Behavioral Disaffection (N = 89)	2.54 (0.71)	2.39 (0.74)	2.47 (0.73)	*t* test	0.33
EvsD – Emotional Disaffection (N = 89)	2.67 (0.91)	2.58 (0.70)	2.63 (0.82)	*t* test	0.62

This uses the sample of child participants included in the effectiveness analyzes.

At baseline, the mean T score on the child-report MASC-2 was 58.5 (SD = 12.2), and the mean T score on the parent-report MASC-2 was 62.2 (SD = 15.8) for the sample as a whole, which is in the High Average range. The mean EvsD subscale scores ranged from 2.47 (SD = 0.73) for Behavioral Disaffection to 3.36 (SD = 0.62) for Behavioral Engagement for the sample as a whole. There were no significant differences in baseline scores between groups.

#### Treatment effectiveness

Results from the mixed effects modeling approach that was used to estimate pre- to post- mean change scores and its 95% CI for student outcomes between the two conditions are shown in Table 2. The mean change score for the child-reported MASC-2 was 1.85 (SE = 1.72) points in the FRIENDS condition and 2.92 (SE = 1.73) points in the CATS condition; the mean difference between groups was 1.06 (SE = 2.43). The 95% CI was [−4.08, 6.21] and included Zero indicating that the estimated differences between condition are similar in their treatment effects. Similarly, the mean change score for the parent-report MASC-2 was 2.31 (SE = 2.75) points in the FRIENDS condition and 2.25 (SE = 2.25) points in the CATS condition. The mean difference in change score for the reported parent report MASC-2 between the two conditions was 0.06 (SE = 3.68) and the 95% CI was [−7.03, 11.58], indicating that the estimated differences between conditions are similar in their treatment effects.

**Table 2:** Change (Pre minus Post) scores for student outcomes.

Outcomes	FRIENDS *N* = 48 students and 9 schools		CATS *N* = 43 students and 10 schools		Difference between the Groups				
Change scores (pre minus post)	Mean	SE	Mean	SE	Mean	SE	95% CI lower	95% CI upper	*P*r > t
Child-Report MASC-2 (*N* = 91)	1.85	1.72	2.92	1.73	1.06	2.43	−4.08	6.21	0.667
Parent-Report MASC-2 (*N* = 36)	2.31	2.75	2.25	2.25	0.06	3.68	−7.43	7.55	0.987
EvsD – Behavioral Engagement (*N* = 87)	0.06	0.08	0.04	0.08	−0.02	0.12	−0.27	0.23	0.887
EvsD – Emotional Engagement (*N* = 87)	−0.02	0.12	0.07	0.12	0.09	0.17	−0.27	0.45	0.612
EvsD – Behavioral Disaffection (*N* = 87)	0.05	0.09	−0.06	0.09	−0.10	0.13	−0.37	0.17	0.430
EvsD – Emotional Disaffection (*N* = 87)	0.04	0.10	−0.16	0.10	−0.20	0.14	−0.47	0.08	0.159

Students (*N* = 91) were clustered within schools (*N* = 19 schools). Due to the cluster-randomized design, students were eliminated from the analysis for each individual outcome measure if there were not 3 students from within their school with baseline- and post- assessment data on that specific outcome measure.

For the engagement and disaffection subscales the mean change scores ranged from −0.08 (SE = 0.61) to 0.28 (SE = 0.42) for FRIENDS and from −0.81 (SE = 0.50) to 0.36 (SE = 0.60) for CATS. The mean difference in change scores between the conditions ranged from −1.0 (SD = 0.69) to 0.44 (SD = −1.36), and each of the 95% CIs included Zero.

In sum, the estimated 95% CIs of mean pre-to-post changes between the two treatments indicated that the two treatment conditions (i.e., CATS and FRIENDS) produced similar treatment effects for child-reported anxiety symptoms, parent-reported anxiety symptoms, and teacher-reported engagement and disaffection. The estimated 95% CI showed a wide range in the treatment effects of both treatments. Students who received either intervention experienced similar improvements; however, CATS used fewer sessions (8 sessions total) vs. FRIENDS (12 sessions total).

### Implementation outcomes

#### Dosage

Of the 75 students who were randomized to the FRIENDS condition, 59 (79%) received at least 1 session of the FRIENDS group, 37 (49%) received at least six sessions (i.e., at least half of the total sessions), and five (7%) received all 12 sessions. Of the 83 students who were randomized to the CATS condition, 61 (73%) received at least 1 session of the CATS group, 48 (58%) received at least four sessions (i.e., at least half of the total sessions), and 26 (31%) received all eight sessions.

#### Fidelity to group content

The mean content fidelity score for sessions in the FRIENDS condition was 0.89 (*SD* = 0.09; range [0.75, 1.0]), and the mean content fidelity score for sessions in the CATS condition was 0.89 [SD = 0.12; range (0.60, 1.0)]. This indicates that the intervention was delivered with acceptable fidelity in both conditions.

#### Cost and cost-effectiveness

On average, therapists leading FRIENDS cohorts spent 1.7 h per week (SD = 1.1) doing so; those leading CATS cohorts spent 1.9 h (SD = 1.2). Supervisors for FRIENDS cohorts spent 2.3 h per week (SD = 1.5) doing so, while those for CATS spent 1.4 h (SD = 0.9).

Due to the difference in the number of weeks for the FRIENDS protocol (i.e., 12 weeks) compared to the CATS (i.e., 8 weeks) as well as differences in the time spent during the week prior to initiation of the groups, total therapist time per cohort was 21.9 h (SD = 14.1) for FRIENDS and 16.5 h (SD = 11.0) for CATS. The 5.4-h difference in therapist time between groups was not statistically significant (*p* = 0.31). Total supervisor time per cohort was 29.5 h (SD = 17.8 h) for FRIENDS and 12.2 h (SD = 7.4) for CATS. The 17.3-h difference in supervisor time between groups (SE = 3.5) was statistically significant (*p* < 0.0001, 95% CI: 10.3-to-24.3-h reduction). The 22.7-h reduction (SE = 7.2) of combined therapist and supervisor time was statistically significant (*p* = 0.002, 95% CI: 8.6 to 36.8 h).

Translating hours into costs, the sums of the average costs for therapists and supervisors were $1983 for FRIENDS and $1,092 for CATS. The difference between conditions was -$891 (SE = 278), which was statistically significant (*p* = 0.001, 95% CI, 346 to 1,436). Taken together, the results of the cost analyzes indicate that the cost of delivering a CATS group was almost $900 less than the cost of delivering a FRIENDS group.

Among our sample of 83 students, the average improvement (i.e., reduction) in child-report MASC-2 scores was 1.09 (SE = 2.34) which was not statistically significant (*p* = 0.64, 95% CI: −3.50 to 5.58 improvement). The correlation of the differences in costs and effects was −0.07.

**Figure 1:**
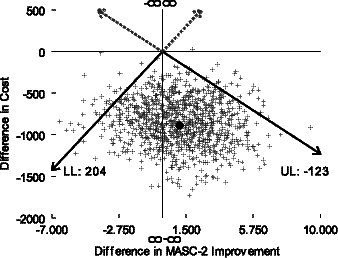
Cost-effectiveness plane. Cost-effectiveness plane indicates that CATS tends to cost less and improve average child self-report MASC-2 score more than FRIENDS.

**Figure 2:**
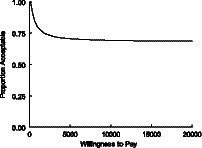
Cost-effectiveness acceptability curve. Cost-effectiveness acceptability curve indicates that, compared to FRIENDS, CATS has at least a 68% chance of being good value for all values of willingness to pay for a one point improvement in average child self-report MASC-2 score more between 0 and $10,000.

The point estimate for the cost-effectiveness ratio indicates CATS saved -$817 (−891/1.09) per one point improvement in a cohort’s average child self-report MASC-2 score (i.e., CATS had lower costs and greater effectiveness–dominated–FRIENDS). Figure 1 shows the empirical distribution of the differences in cost and effect on the cost-effectiveness plane. The fact that the point estimate (black circle) lies in the lower right quadrant indicates the dominance relationship. However, the mass of points in the lower left quadrant indicates that we cannot rule out that FRIENDS may increase both costs and effects. Due to this mass, the 95% confidence interval indicates that if our willingness to pay for a 1-point improvement in a cohort’s average self-report MASC-2 score is less than $204, we can be 95% confident of CATS’ value. If it is greater than $204 per 1-point improvement, we cannot be 95% confident. Finally, Figure 2 shows the cost-effectiveness acceptability curve. Based on the curve, for all values of willingness to pay between 0 and 10,000 for a 1-point improvement there is at least a 68% chance that CATS is good value. These types of results are typically interpreted as evidence that CATS is good value.

#### Appropriateness

Across the CATS and FRIENDS group, therapists and supervisors had mixed perspectives about the appropriateness of the intervention for the students in their group. However, compared to therapists and supervisors in the CATS condition, therapists and supervisors in the FRIENDS condition more strongly described aspects of the intervention that were not appropriate for their context and in need of more extensive adaptations.

In the CATS group, a few participants noted that they believed the curriculum was at an appropriate level and was accessible for the students in their groups. Other participants shared that they thought the content of the CATS intervention, including the pacing and amount of material covered and the reading level of the student workbook, was too challenging and not accessible for the students in their group. Additionally, some concerns arose about the contextual appropriateness of the examples used in the CATS intervention, but these few concerns were not described in strong terms. For example, the CATS therapist who brought up this concern simply said, “Some of the examples just kind of were not some things they could relate to” (T42060). The CATS supervisor (S22015) that brought this up focused on one singular word that was used in the curriculum which caused confusion and distracted students.

In contrast, FRIENDS therapists and supervisors more strongly voiced that the intervention needed adaptations to make it more relevant for the students in their groups.

One supervisor spoke about how the curriculum needed to be adapted to be relevant to the children: “It’s culturally tone deaf… as far as dealing with our, our kids. And so we have to make a lot of modifications and changes, and… I don’t know, I think our clinician I worked with did a very good job, as best as she could…” (S31025). A therapist similarly stated, “It felt like we were a different demographic than what this manual was designed for” (T32034).

Another therapist also spoke about how the content of the intervention was not relevant to the children in their group. However, this therapist spoke more positively about the intervention’s adaptability:

*“The other thing that I did not like about the program is that it’s based on children…based on children that are out of the country, not based on the environment, or the culture that I basically look for. However that’s just the book so it’s not such a big negative. The point is that I can change it, its flexible, and make it geared towards the kids that I am working with.”* (T22021)

Another therapist echoed this sentiment, stating, “It took a lot of editing and adjustments.” (T32026).

Importantly, one FRIENDS therapist reported a different perspective about the intervention, sharing that they thought that the curriculum’s scenarios and terminology were relatable:

*“So I do love the fact the activities were, actually relatable, I found that the participants could really relate to the characters within the activities, they could relate to the scenarios.”* (T12012)

## Discussion

The current study examined the contextual appropriateness, effectiveness and cost-effectiveness of a group CBT intervention with adaptations to improve cultural fit (i.e., CATS) delivered by school-based therapists with Train-the-Trainer implementation support in urban schools. Results generally supported the promise of the CATS intervention in this context, as it achieved equivalent results with fewer sessions, was more cost-effective, and was viewed as more contextually appropriate. However, the reduction in child-reported anxiety symptoms was small for both interventions. This adds to the literature on the implementation of CBT for anxiety in community settings by examining the outcomes of an adapted group CBT protocol following real-world implementation.

Quantitative results indicated that student outcomes were equivalent for students in schools randomized to the CATS and the FRIENDS condition, suggesting that the adaptations made to CATS (including a reduction in the total number of sessions from 12 to 8) did not result in reduced effectiveness. Specifically, the difference in change scores between group did not surpass the predefined margin of error of 4.2 for any outcomes indicating that there were no clinically important differences in effectiveness between the groups. Prior research has shown that a 12-week group CBT intervention for social anxiety delivered by trained school counselors led to reduction in student anxiety symptoms compared to a control condition, and also led to outcomes that were not significantly worse than obtained by the same intervention delivered by clinical psychologists with experience in CBT for pediatric anxiety (37). The current results add to this literature by providing empirical evidence that the CATS group anxiety manual shows the same effectiveness as an established program, when both interventions were delivered by school-based therapists.

Students in schools randomized to the FRIENDS condition showed an average improvement of nearly 2 points on the child-report MASC-2 and just over 2 points on the parent-report MASC-2, and those in schools randomized to the CATS condition showed an average improvement of nearly 3 points on the child-report MASC-2 and just over 2 points on the parent-report MASC-2. These improvements are relatively small. Moreover, teacher-report engagement and disaffection scores remained stable across time. Because there was not a no-treatment control group, within-group change scores should be interpreted with caution; nevertheless, these results are consistent with the reduction in effectiveness when interventions are implemented in real-world settings that is often seen in the literature (38, 39). The small within-group change scores are also consistent with findings that prevention programs with low acuity samples tend to yield small effect sizes, compared to interventions with high acuity samples, although prevention programs implemented at scale have the potential for meaningful population-level improvements (40).

Because the CATS intervention had four fewer sessions compared to FRIENDS (and to a lesser extent because CATS nominally took fewer hours per session), average costs to implement an 8-session cohort were $891 (SE = 278, *p* = 0.001; 95% CI, 346 to 1,436) lower than those for a FRIENDS 12-session cohort. CATS incremental cost-effectiveness ratio was -$817 (−891/1.09) per one point improvement in a group’s average child self-report MASC-2 score, indicating it dominated FRIENDS. Based on the acceptability curve, there’s at least a 68% chance that CATS is good value for all values of willingness to pay between 0 and $10,000. These results add to the nascent literature [e.g., (41)] about cost- and cost-effectiveness of mental health interventions by providing a direct comparison of two school-based interventions.

Qualitative results were consistent with the adapted intervention (i.e., CATS) resulting in improved contextual appropriateness for the United States urban school context, compared to the un-adapted intervention (i.e., FRIENDS). Although therapists and supervisors in both groups had mixed perspectives about the appropriateness of the intervention for the students in their group (e.g., some concerns about pacing and reading level), therapists and supervisors in the FRIENDS conditions more strongly voiced that the intervention needed to be adapted for cultural relevance. This suggests that the adaptations made to the CATS intervention were helpful, although additional adaptations may still be needed, consistent with the idea that making cultural adaptations to evidence-based interventions is an iterative, ongoing process, and that additional adaptations for specific subgroups of consumers or program delivery staff may be needed (42).

### Limitations and future directions

We note several limitations. First, group interventions were halted prematurely in March of 2020 due to the COVID-19 pandemic and associated school closures, and the project was unable to continue during the subsequent school year, when schools continued to operate primarily virtually. The research team attempted to collect post- data for students whose groups had completed at least half of the planned sessions (i.e., four group sessions for CATS and six group sessions for FRIENDS) at the time of school closures, but this reduced the number of students for whom groups were completed and data were available, and the context of the pandemic may have affected outcome data. Second, even prior to the COVID pandemic, there was considerable missing data for the parent-report outcome. Moreover, some students in both conditions did not receive the planned intervention dosage, due to student absences or therapists being unable to lead each planned session due to time constraints. These implementation challenges are common in real-world implementation, particularly in the context of under-resourced schools (43), and there is no reason to believe that they had a differential effect between the two treatment groups; nevertheless, treatment dosage may have impacted the overall effects observed. Relatedly, the number of students participating and group cohorts completed varied considerably between schools. Finally, it is important to note that therapists and supervisors were assigned to their roles for the purposes of this study, but study supervisors were not necessarily functioning as supervisors outside of the study context, which may have impacted the effectiveness of supervision in both conditions.

Given these limitations, it is important for future research to continue to identify implementation strategies that can successfully support the delivery of group anxiety interventions in school contexts. Factors across the outer setting (e.g., broader social and political context), inner setting (e.g., school or agency leadership support), individual provider (e.g., motivation, training), and the intervention itself (e.g., fit, usability) may influence implementation (44). The current results suggest that adaptations to the group CBT intervention led to improved contextual appropriateness and reduced cost, without a loss of effectiveness. However, future research should examine whether further adaptations would make the intervention more usable in this context, and should continue to develop implementation strategies that are feasible and sustainable in school contexts. The current limitations also highlight the importance of continuing to develop pragmatic research strategies to support data collection in real-world contexts (45).

### Implications and conclusion

These results have important implications for the dissemination of group CBT for anxiety to the urban school context. Results overall support the promise of abbreviating and adapting an evidence-based CBT group intervention to improve contextual appropriateness, cultural fit, and cost-effectiveness. Specifically, results support the use of the CATS group intervention, as compared to evidence-based group CBT interventions for anxiety, in the urban school context. The results also suggest that this intervention can be delivered by school-based therapists using a train-the-trainer model of implementation support. However, the small change scores for student-reported anxiety symptoms and parent-reported anxiety symptoms in both groups, as well as the variability in observed dosage, suggest that it may be important to use additional implementation strategies to address implementation challenges in the context of under-resourced schools. This is a key next step to address the critical challenge of identifying prevention and treatment models for youth anxiety, as well as associated implementation approaches, that are effective, feasible, contextually appropriate, and cost-effective to implement in usual care.

## Data availability statement

The raw data supporting the conclusions of this article will be made available by the authors, without undue reservation.

## Ethics statement

The studies involving human participants were reviewed and approved by Institutional Review Board (IRB) of the Philadelphia Department of Public Health. Written informed consent to participate in this study was provided by the participants’ legal guardian/next of kin.

## Author contributions

RE is the principal investigator for the study, generated the idea and designed the study, and takes responsibility for data collection and the integrity of the data. GL drafted the manuscript, and approved all edits. AJ, MK, and RB collaborated on the design of the study. AJ prepared all quantitative analyzes. HG prepared the cost analyzes. RC, JG, SB-D, and GL contributed to qualitative analyzes. QR-M and TW coordinated the study and have contributed to the design of the study. All authors contributed to the article and approved the submitted version, and reviewed and provided feedback for the manuscript.

## Funding

The study was funded by the National Institute of Mental Health (NIMH), R01MH108555 to RE (PI). Additionally, the National Institute of Mental Health (NIMH), K23MH122577 to GL supported the time of the first author preparing this manuscript.

## Conflict of interest

RB is principal at Implementation Science & Practice, LLC. She receives royalties from Oxford University Press, consulting fees from United Behavioral Health and OptumLabs, and serves on the advisory boards for Optum Behavioral Health, AIM Youth Mental Health Foundation, and the Klingenstein Third Generation Foundation outside of the submitted work.

The remaining authors declare that the research was conducted in the absence of any commercial or financial relationships that could be construed as a potential conflict of interest.

## Publisher’s note

All claims expressed in this article are solely those of the authors and do not necessarily represent those of their affiliated organizations, or those of the publisher, the editors and the reviewers. Any product that may be evaluated in this article, or claim that may be made by its manufacturer, is not guaranteed or endorsed by the publisher.
